# Quality Assessment of Smartphone Medication Management Apps in France: Systematic Search

**DOI:** 10.2196/54866

**Published:** 2024-03-18

**Authors:** Mickael Toïgo, Julie Marc, Maurice Hayot, Lionel Moulis, Francois Carbonnel

**Affiliations:** 1 Department of General Practice Univ Montpellier Montpellier France; 2 PhyMedExp Univ Montpellier, CNRS, INSERM, CHU Montpellier Montpellier France; 3 Clinical Research and Epidemiology Unit Department of Public Health Univ Montpellier, CHU Montpellier Montpellier France; 4 Pathogenesis and Control of Chronic and Emerging Infections Univ Montpellier, INSERM, EFS, University of Antilles Montpellier France; 5 Desbrest Institute of Epidemiology and Public Health Univ Montpellier, INSERM Montpellier France

**Keywords:** medication adherence, mobile apps, telemedicine, reminder system, behavioral therapy, mHealth, mobile health, app, apps, applications, smartphone apps, medication, medications, adherence, search, searches, searching, systematic, App Store, Google Play, French, reminder, reminders, MARS, quality, Mobile App Rating Scale, mobile phone

## Abstract

**Background:**

Adherence to medication is estimated to be around 50% for chronically ill patients in high-income countries. Improving the effectiveness of adherence interventions could have a far greater impact on population health than any improvement in specific medical treatments. Mobile health (mHealth) is one of the most effective solutions for helping patients improve their medication intake, notably through the use of mobile apps with reminder systems. With more than 327,000 apps available in the mHealth field, it is difficult for health care professionals and patients alike to choose which apps to recommend and use.

**Objective:**

We aim to carry out a systematic search of medication management smartphone apps available in France that send reminders to patients and assess their quality using a validated scale.

**Methods:**

Mobile apps were identified in October and November 2022 after a systematic keyword search on the 2 main app download platforms: App Store (Apple Inc) and Google Play Store. Inclusion criteria were free availability, date of last update, and availability in French. Next, 2 health care professionals independently evaluated the included apps using the French version of the Mobile App Rating Scale (MARS-F), an objective scoring system validated for assessing the overall quality of apps in the mHealth field. An intraclass correlation coefficient was calculated to determine interrater reliability.

**Results:**

In total, 960 apps were identified and 49 were selected (25 from the App Store and 24 from the Google Play Store). Interrater reliability was excellent (intraclass correlation coefficient 0.92; 95% CI 0.87-0.95; *P*<.001). The average MARS-F score was 3.56 (SD 0.49) for apps on the App Store and 3.51 (SD 0.46) for those on the Google Play Store, with 10 apps scoring above 4 out of 5. Further, 2 apps were tested in at least one randomized controlled trial and showed positive results. The 2 apps with the highest ratings were *Mediteo rappel de médicaments* (Mediteo GmbH) and *TOM rappel medicaments, pilule* (Innovation6 GmbH), available on both platforms. Each app’s MARS-F score was weakly correlated with user ratings on the App Store and moderately correlated on the Google Play Store.

**Conclusions:**

To our knowledge, this is the first study that used a validated scoring system to evaluate medication management apps that send medication reminders. The quality of the apps was heterogeneous, with only 2 having been studied in a randomized controlled trial with positive results. The evaluation of apps in real-life conditions by patients is necessary to determine their acceptability and effectiveness. Certification of apps is also essential to help health care professionals and patients identify validated apps.

## Introduction

Therapeutic adherence is defined by the World Health Organization as “the extent to which the behaviors of a person required to take medication, follow a diet and/or change lifestyle correspond to the recommendations agreed with a healthcare professional” [1]. It is estimated to be around 50% for people with chronic diseases in high-income countries [1]. The result is an increased risk of complications, hospitalization, and mortality for these patients, as well as consequently higher health care costs [2-5]. Improving the effectiveness of adherence interventions could potentially have a far greater impact on population health than any improvement in specific medical treatments [1].

Mobile health (mHealth) is a promising strategy to optimize therapeutic adherence [6]. mHealth covers medical and public health practices based on mobile devices such as cell phones, patient monitoring systems, personal digital assistants, and other wireless devices [7]. It is dominated by the use of health and wellness smartphone apps, the number of which continues to grow yearly [8]. In 2021, over 327,000 mHealth apps were listed in digital stores [8].

In France, almost 90% of French people aged older than 12 years owned a smartphone in 2022 and 72% of them had downloaded apps [9]. For their part, French general practitioners seem to be in favor of mHealth apps or devices and ready to prescribe these in their practice, but at the same time stress the importance of validating the use of these apps through randomized clinical trials and certification by health care professionals [10].

Several studies have confirmed that the use of a medication management app improves adherence to therapy, notably by sending reminders, even in older patients with no experience of using new technologies [11-14]. However, no study evaluating the quality of medication management apps via a validated score has been published to date.

The main objective of this study was to identify medication management apps to improve patient adherence and assess their quality, using a validated scale. The secondary objective of this study was to identify high-quality medication reminder apps and to provide recommendations to any patient needing to take one or more medications, regardless of pathology.

## Methods

### Overview

This involved a systematic search of smartphone apps with content evaluation, carried out between October 1, 2022, and June 20, 2023. It was reported in line with the PRISMA (Preferred Reporting Items for Systematic Reviews and Meta-Analyses) checklist for reporting a systematic review (items not pertinent to a systematic search of apps were considered not applicable) [15].

### App Selection

Apps were searched from October 1 to November 30, 2022, on the App Store and Google Play Store. These 2 platforms were used by over 99% of mobile users to download apps in 2022 [16].

The keywords searched on the download platforms were “medication reminder” (“rappel de médicament”), “medication monitoring” (“suivi de médicament”), “treatment reminder” (“rappel de traitement”), “treatment monitoring” (“suivi de traitement”), and “pillbox” (“pilulier”). The selection of search terms was based on existing studies, which were adapted after several prior search attempts on digital download platforms [17]. We also included native health apps, automatically present on iOS and Android phones without the need to download them. Being preinstalled on smartphones, they cannot be identified by our search equations on app stores. However, they are available and easily accessible to users. Android currently has no native health app.

Inclusion criteria were as follows: (1) medication reminder app; (2) availability in the French language; (3) free (apps free for only a trial period were not selected); (4) updated within the last 2 years (since 2022, Google Play Store removes apps without updates for more than 2 years [18], and for the App Store, this period is 3 years for apps with few downloads in the last 12 months [19]); and (5) not specific to a single treatment (eg, the contraceptive pill) or to the treatment of a particular pathology. Inclusion criteria were based on the data available on the apps’ presentation page, that is, the title, description, images, and general information about the app. Apps were excluded after download in the event of (1) or (2) unavailability on download platforms at the time of evaluation by one of the evaluators.

The decision to assess exclusively free apps was grounded in the primary target audience of patients encountering diverse and, at times, multiple impediments to consistent medication adherence. The high cost of an app is one of the main reasons why it is not downloaded [20]. In addition, users frequently report hidden costs as the main reason for discontinuation [20]. So, considering the price as a dissuading factor for the adoption of health apps, proposing a paid app to this population appeared inadequate [21].

### Evaluation via the French Version of the Mobile App Rating Scale App

The Mobile App Rating Scale (MARS) was used to evaluate the apps. This score was previously validated for the overall quality assessment of an app in the field of mHealth [22]. The French version of this score, MARS-F, has also been validated for use with French-language apps [23].

MARS-F is a 19-item questionnaire divided into 4 objective evaluation sections (A to D): engagement (5 items: entertainment, interest, personalization, interactivity, and adaptation to target group), functionality (4 items: app performance, ease of use, navigation, and app gesture design), aesthetics (3 items: layout, graphic design, and overall visual appeal), and information (7 items: accuracy of app description, precise app objectives, quality of information, quantity of information, visual information, credibility of information, and scientific evidence; Multimedia Appendix 1). Each item is rated with a Likert scale from 1 (“inadequate”) to 5 (“excellent”). When specific objectives (item 14) were not delineated, and pertinent information (items 15, 16, and 17) or scientific evidence (item 19) was lacking, the respective items were rated as “not applicable.” Consequently, these items were excluded from consideration in the overall scoring process. In total, MARS-F is a score out of 5, corresponding to the average of these 4 sections. Section E, not included in the overall MARS-F calculation, evaluates the apps’ subjective quality via 4 items and is described separately. The MARS-F also includes a preliminary descriptive section on the apps’ characteristics, including users’ ratings on the platforms, the apps’ target objectives, the strategies used, its affiliation (commercial, governmental, and academic), as well as the technical aspect (password protection, possible sharing, and internet access required).

In total, 2 family medicine residents evaluated each app independently. Before starting, they assisted with a training video on the use of MARS-F and trained in real-life conditions by evaluating 3 apps not selected for this study. The scores for each item were then discussed one by one to ensure a similar understanding on the part of the 2 evaluators.

Apps available on the 2 platforms were evaluated independently on iOS and Android. Each app was tested and used for at least 15 minutes. Evaluations took place from February to June 2023.

### Statistical Analysis

App characteristics were described using frequencies and proportions. To determine interrater reliability, a 2-way mixed-effects model intraclass correlation coefficient (ICC) was calculated for the mean of the raters. An ICC below 0.5 was considered poor, moderate between 0.5 and 0.75, good between 0.75 and 0.90, and excellent if above 0.90 [24].

The average of the scores given by the 2 raters or evaluators was used for the final rating of each app. Apps were compared according to their respective MARS-F quality score. The results were presented as mean (SD) and median and quartiles.

The correlation between the average rating of app users on download platforms and the MARS-F obtained was measured by Spearman correlation after a normality test. Regarding the correlation between MARS-F and user ratings, we excluded apps with a limited number of ratings on platforms, as they may not be highly representative of users. The choice of the threshold was determined following a sensitivity analysis, wherein the correlation was computed for various thresholds: five ratings on platforms, 10, 20, 30, 50, and so on. The selected threshold was the lowest one for which both the lower and upper thresholds yielded similar results. The threshold of 20 user ratings was finally selected. The correlation between reviewers’ subjective assessment of the apps via item 23 (“What is your overall star rating for the application?”) and the MARS-F obtained for each app was also calculated by Pearson correlation after a normality test. The correlation was judged as very strong from 1 to 0.9, strong from 0.9 to 0.7, moderate from 0.7 to 0.5, weak from 0.5 to 0.3, and very weak from 0.3 to 0.

All statistical analyses were performed with EasyMedStat (version 3.29; EasyMedStat).

### Ethical Considerations

The Research Ethics Committee of the University of Montpellier approved this research project (UM 2022-006bis; Multimedia Appendix 2). This study was not funded.

## Results

### App Selection

**Figure 1:**
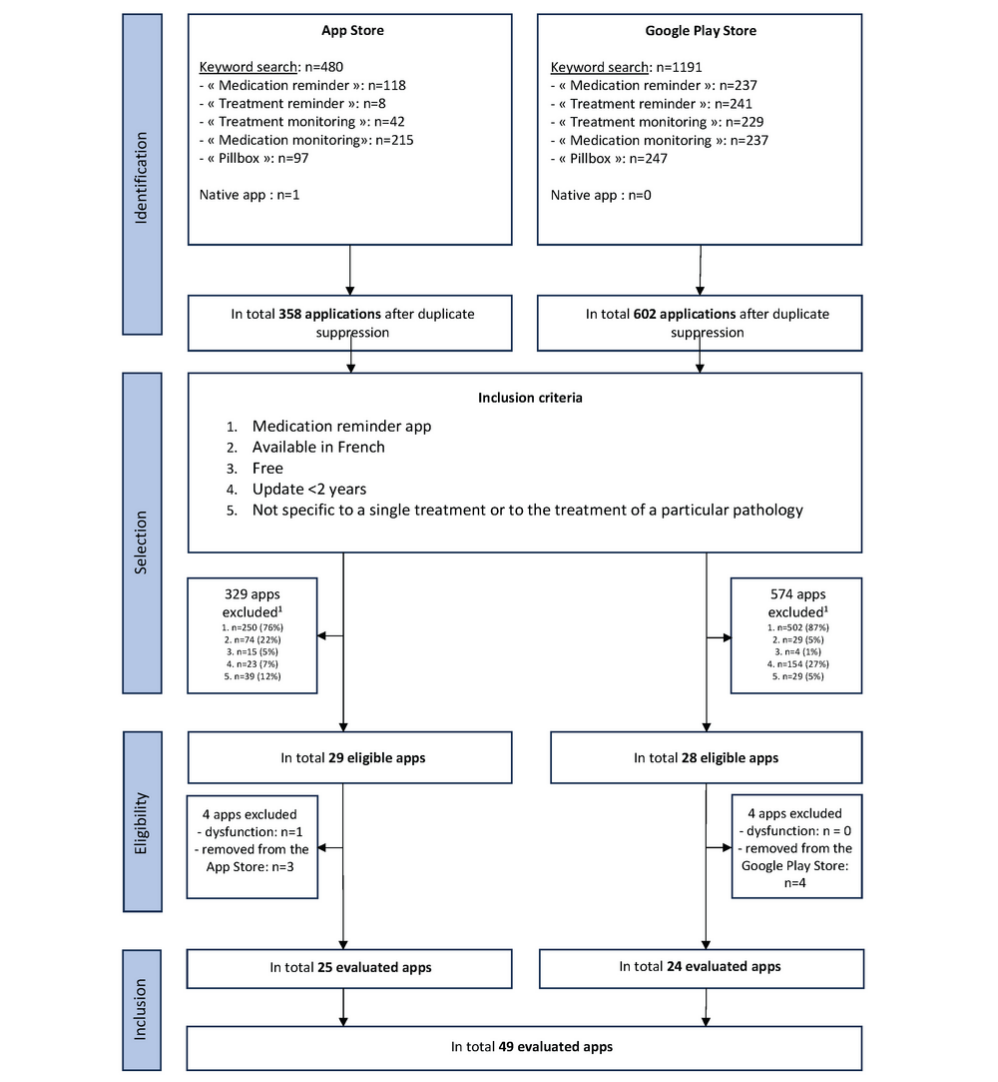
Flowchart showing app selection. The numbers under “selection” refer to the inclusion criteria. The sum of the percentages exceeds 100% because a single app may not meet different inclusion criteria.

After a keyword search and the addition of native apps, 480 apps were identified on the App Store and 1191 on the Google Play Store. A total of 25 apps meeting the inclusion and exclusion criteria were selected from the App Store and 24 from the Google Play Store (Figure 1).

### Description of the Apps

In total, 51% (n=25) of the apps evaluated were available on the App Store and 49% (n=24) on the Google Play Store, 10 of which were common and available on both platforms. Further, 40 had been rated by users on both platforms (23 on the App Store and 17 on the Google Play Store).

Although all the apps were available free of charge, some had a paid version, as in the case of 15 (60%) App Store apps and 12 (50%) Google Play Store apps. The affiliation of the developers was not always known, but the majority were commercial (24/25, 96% on the App Store; 21/24, 87.5% on the Google Play Store).

Technical aspects according to the download platform are summarized in Table 1.

**Table 1:** Technical aspects of apps according to platform.

	App Store (n=25), n (%)	Google Play (n=24), n (%)
Allows sharing	12 (48)	10 (42)
Has an app-linked community	3 (12)	1 (4)
Has the ability to password-protect	10 (40)	3 (12.5)
Requires a login ID	4 (16)	2 (8)
Sends reminders	25 (100)	24 (100)
Needs internet access to work	10 (40)	8 (33)

### Evaluation Reliability

MARS-F interrater reliability for all apps was excellent (ICC 0.92; 95% CI 0.87-0.95; *P*<.001).

For each section, interrater reliability was excellent for engagement (ICC 0.92; 95% CI 0.88-0.95; *P*<.001) and for subjective app quality (ICC 0.95; 95% CI 0.92-0.97; *P*<.001). It was good for aesthetics (ICC 0.81; 95% CI 0.70-0.89; *P*<.001) and the information section (ICC 0.86; 95% CI 0.78-0.92; *P*<.001). Finally, interrater reliability was only moderate for the functionality part (ICC 0.70; 95% CI 0.52-0.82; *P*<.001).

### Quality of Apps Based on MARS-F

The mean MARS-F score was 3.56 (SD 0.49) for iOS apps and 3.51 (SD 0.46) for Android apps (Table 2). The full set of scores by the app is presented in Multimedia Appendix 3.

**Table 2:** Average score, range, and median per section.

Variable	iOS (n=25)			Android (n=24)	
	Score, mean (SD)	Score, median (Q1^a^-Q3)	Score, mean (SD)		Score, median (Q1-Q3)
Section A: engagement	3.19 (0.616)	3.3 (2.8-3.6)	3.15 (0.628)		3.15 (2.8-3.52)
Section B: functionality	4.26 (0.448)	4.38 (4-4.62)	4.17 (0.431)		4.25 (3.84-4.53)
Section C: aesthetics	3.5(0.555)	3.67 (3.17-4)	3.38 (0.599)		3.5 (3-3.83)
Section D: information	3.29 (0.708)	3.3 (2.83-3.8)	3.33 (0.561)		3.17 (2.83-3.81)
MARS-F^b^: global quality	3.56 (0.485)	3.6 (3.21-3.98)	3.51 (0.455)		3.48 (3.29-3.88)
Section E: subjective quality	2.52 (1.12)	2.38 (1.5-3.62)	2.55 (1.19)		2.25 (1.72-3.22)

^a^Q: quartile.

^b^MARS-F: Mobile App Rating Scale, French version.

### General App Features

The iOS apps with the highest MARS-F scores were *TOM rappel medicament, Pilule* (MARS-F score: 4.37; Innovation6 GmbH), then *Mediteo rappels de médicaments* from (MARS-F score: 4.34; Mediteo GmbH), followed by *Rappels de médicaments* from (MARS-F at 4.13; smartpatient GmbH). The Android apps with the highest MARS-F scores were *Mediteo rappels de médicaments* (MARS-F score: 4.32), followed by *TOM Rappel medicaments, pilule* (MARS-F score: 4.24) and *Rappel de pilule et medicament* (MARS-F score: 4.09; Medisafe). The MARS-F ranking of apps by platform is available in Multimedia Appendix 4.

In total, 84% (n=21) of the apps available on the App Store had a MARS-F score above 3, with 5 (20%) scoring above 4. On Google Play Store, 79% (n=19) of apps had a MARS-F score higher than 3, including 5 (21%) with a score higher than 4.

The “scientific evidence” item was completed for 4 (16%) apps on the App Store and 2 (8%) apps on the Google Play Store, 2 of which were common to both platforms.

### Correlation Between MARS-F and User Ratings

In total, 23 (92%) apps were rated by users on the App Store, with an average rating of 4.38/5 (range 2.9-5.0) and an average number of ratings of 475 (range 1-3900; Multimedia Appendix 5). On Google Play Store, 17 (71%) apps were rated by users, with an average score of 4.17/5 (range 3.3-4.8) and an average number of ratings of 25,062 (range 24-223,000).

After performing sensitivity analyses, tests looking for a correlation between a given app’s MARS-F and average user rating were carried out on apps with at least 20 reviews on the platforms. The correlation was weak on the App Store (ρ=0.46; *P*=.12) and moderate on the Google Play Store (ρ=0.55; *P*=.02).

### Correlation Between MARS-F and Reviewers’ Subjective Evaluation

The correlation between MARS-F and item 23 (“What is your overall star rating of the app?”) was considered very strong for iOS apps (ρ=0.93; *P*<.001) and strong for Android apps (ρ=0.88; *P*<.001).

The mean score for this item was 2.90 and 2.98 on iOS and Android, respectively, which is below the respective mean MARS-F.

## Discussion

### Principal Results

The main objective of our study was to identify medication management apps to improve patient adherence. In total, 960 apps were identified: 358 (37.3%) on App Store and 602 (62.7%) on Google Play Store. This imbalance in favor of the Google Play Store has already been observed in several studies [25,26]. However, after selection, the number of apps was balanced 51% (n=25) on the App Store and 49% (n=24) on the Google Play Store, so it seems that the Google Play Store search engine offers more apps for the same keyword. This balanced proportion is consistent with a 2019 medication management app evaluation study in which 328 apps available in English were studied [17]. In this study, 53.4% and 46.6% of apps were retrieved from the Google Play Store and the App Store, respectively [17]. Our study evaluated the content of these apps according to the information available on app download stores without downloading them.

Our study highlighted a cybersecurity issue: only 40% (n=10) of apps identified on iOS had password-protected access, and 4% (n=1) required a login ID. These figures were even lower for Android apps, at 12.5% (n=3) and 8.3% (n=2), respectively. This was also the case for pain management apps, with 39% of apps evaluated allowing password protection and 44% requiring login [27].

The overall quality of the medication reminder apps evaluated is considered acceptable to moderate, with 84% (n=21) of apps on the App Store achieving a MARS-F higher than 3/5 with an average of 3.56 (SD 0.49) and 79% (n=19) on Google Play Store with an average MARS-F of 3.51 (SD 0.46). We have no point of comparison in the absence of any previous French-speaking or English-speaking study evaluating medication reminder apps by a validated score. Nevertheless, these results were expected, as they are consistent with other French studies on health apps for smoking cessation (mean MARS-F of 3.49, SD 0.57) for 14 apps [28], nutrition (mean MARS-F of 3.26, SD 0.43) for 15 apps [29], mental health (mean MARS-F of 3.16, SD 0.43) for 12 apps [30], or oral hygiene (mean MARS-F of 3.20, SD 0.38) for 9 apps [31].

All the other studies reported that the functionality section of the MARS scale had the highest ratings, which was also the case in our study, with an average of 4.26 (SD 0.45) for the App Store and 4.17 (SD 0.43) for the Google Play Store for this section. This shows that most apps are functional, which also coincides with the fact that only 1 app was excluded for malfunction in our study. In contrast, the section with the lowest average score was the engagement section (average of 3.19, SD 0.62, on the App Store and 3.15, SD 0.63, on the Google Play Store), followed by the information section (average of 3.29, SD 0.71, and 3.33, SD 0.56, on the App Store and Google Play Store, respectively), in line with the literature [32-37]. Several other studies have noted lower scores for the information section [25,27,30,31,38-41]. Nevertheless, these 2 sections are regularly cited as being those for which we find lower averages. Further, 1 exception is the study by Salehinejad et al [42], for which the highest-rated section was the information section. This is a special case, as the evaluation was concerned with COVID-19 management apps, which were probably created for information purposes in the first place.

About the information section, the lack of involvement from health care professionals, universities, or governmental organizations, may explain the observed low scores, thereby lowering the credibility of the apps. This point is assessed in the MARS-F by item 18 (“Does the application come from a legitimate source (specified in the application store description or in the application itself)?”). Often difficult to ascertain [16], most apps were affiliated with a commercial company, with only 14.6% of medication management apps and 15.2% of apps for patients with genitourinary tumors showing involvement by health care professionals [43]. Involvement by health care professionals was sometimes mentioned on the apps’ websites, but without explaining their precise role or degree of involvement. The “scientific evidence” item (item 19) was completed for 4 different apps out of the 49 evaluated in our study, 2 of which were common to both the App Store and Google Play Store. These 2 apps are the only ones to have been tested in at least one randomized controlled trial with positive results: smartpatient GmbH or MyTherapy’s *Rappels de médicaments* [44] and Medisafe’s *Rappel de pilule et medicament* [45]. They are ranked among the best apps on the 2 platforms according to their MARS-F obtained in this study. This lack of scientific validation of health care apps is a recurring theme [25,29,32,33,37].

In our study, user ratings of digital app stores were higher than MARS-F, as expected [27,29]. The absence or low correlation between user rating and MARS score has also been described previously [33,46]. To our knowledge, only the study by Chen et al [47] found a link between the quality of apps assessed by the MARS score and user rating for drug information apps. The average MARS score of apps with higher user star ratings was significantly higher than for apps with lower user star ratings (3.38, SD 0.64, vs 3.05, SD 0.64, *P*<.001) [47]. However, this may be explained by their study design: 3 out of 7 assessors were not health care professionals, which may be a bias.

The correlation between item 23 (“What is your overall star rating for the app?”) and the MARS-F was very strong for apps on iOS and strong for apps on Android. These results are consistent with other studies dealing with nutrition and mental health apps, respectively [29,30]. It is interesting to note that the subjective rating of the reviewers correlated with the overall quality of the app assessed by an objective scale, unlike the user rating. It is challenging to predict whether this is due to the evaluators’ experience or the fact that they have delved deeper into the evaluation of apps using the MARS score.

### Strengths and Limitations

The main limitation of this study is the mobile app sector itself since this study is a reflection of the supply and quality of apps for a specific period. We note, for example, that 6 apps (3 on the App Store and 3 on the Google Play Store) were excluded because they were no longer available a few months after they were identified and downloaded for evaluation. This difficulty had already been highlighted in a study that aimed to evaluate apps for pregnant women at 2-year intervals, in 2017 and then in 2019. One of the things that stood out was that the best app in 2017 was no longer available in 2019 at the time of the second evaluation [40]. The presence of new versions following updates is another element that can influence the quality of an app over time. App selection by only one of the evaluators is also a limitation, as it is possible that some eligible apps have not been identified. In terms of evaluation, the apps were assessed after they had been in use for a minimum of 15 minutes, so additional strengths or weaknesses of each app may not have been detected due to this limited duration.

Our choice to evaluate only free apps is also open to criticism. It could be argued that this choice is induced by a bias of the health care system in which the authors of this paper, who are French, operate. France has indeed the lowest share of out-of-pocket health expenditure among all EU countries [48]. Yet, medication nonadherence is a global problem. Costs attributed to “all causes” nonadherence range from US $5271 to US $52,341 [49]. The high cost of a paid app is dissuasive and hidden costs have been demonstrated as one of the main reasons for discontinuation of the use of an app [20,21]. To our knowledge, the superiority of a paid medication management app over a free one has yet to be demonstrated.

Finally, the MARS score was used because its use is simple, validated, and widespread in the evaluation of health care apps. However, it has several limitations. The first is the absence of data security and privacy evaluation criteria, although the presence of a password and login is indicated in the descriptive section. These points are nevertheless a concern for users of health care apps [50]. The second limitation is the absence of a threshold for judging app quality. We have described it here as moderate, as in several other studies, which found similar average scores, but this was not described when this tool was created [51].

To our knowledge, no other study has evaluated medication management apps using a validated scale. The identification method combined with the inclusion and exclusion criteria enabled an exhaustive analysis of free medication reminder apps available to French patients. These were not selected based on user ratings or the number of downloads from digital stores, which made it possible to evaluate apps that are not promoted on platforms but may nevertheless be relevant to patients. The weak correlation between app quality and user rating found in this study supports this approach. Apps available for iOS and Android devices were evaluated independently on each operating system, as there may be differences in terms of updating or functionality depending on the device used. Finally, the independent testing by 2 evaluators is a strong point, particularly with the observed excellent interevaluator reliability, enabling result confirmation.

### Perspectives

This work is the first step toward facilitating more in-depth studies on top-rated and best-quality apps. App evaluation by patients with the user version of the MARS [52] would be relevant, even though there is currently no validated French version of this scale. The long-term use and benefits of these apps need to be studied in randomized clinical trials, to verify their acceptability and whether or not they improve therapeutic adherence and clinical outcomes in patients undergoing long-term treatment.

The results of this study, added to other studies on app evaluation in the health care field, show the possible ways to improve existing apps and give leads for the creation of new ones. Functionality is paramount, and this point already seems to have been achieved for the majority of apps currently available. The areas that need improvement relate above all to engagement and the information available, of which gamification is 1 avenue to be explored [53]. Data security and privacy protection are also important for patients and should not be neglected.

Currently, France is investing in the use of digital health, particularly for patients with chronic diseases. There is an ongoing project to list more than 50 apps offering exchanges with *Mon Espace Santé* by 2026. Therefore, it is vital to set up certification for existing apps to help doctors in their recommendations [10]. The creation of an app with the help of health care professionals and validated by the Agence du Numérique en Santé would also be a solution that would enable doctors to know which apps to recommend, thus facilitating its use by patients.

## Appendix

Multimedia Appendix 1 Mobile App Rating Scale, French version (MARS-F).

Multimedia Appendix 2 Ethics committee approval.

Multimedia Appendix 3 The full set of scores by app.

Multimedia Appendix 4 Ranking of apps by platform according to the Mobile App Rating Scale, French version (MARS-F).

Multimedia Appendix 5 User ratings and number of evaluation of apps by platform.

Multimedia Appendix 6 PRISMA (Preferred Reporting Items for Systematic Reviews and Meta-Analyses) checklist.

## Abbreviations

ICC: intraclass correlation coefficient
MARS: Mobile App Rating Scale
MARS-F: Mobile App Rating Scale, French version
mHealth: mobile health
PRISMA: Preferred Reporting Items for Systematic Reviews and Meta-Analyses
