# Supplementary material for: Quality Assessment of Smartphone Medication Management Apps in France: Systematic Search
Source: JMIR Mhealth Uhealth. 2024 Mar 18;12:e54866. doi: 10.2196/54866 (PMC10985613; doi:10.2196/54866)
Supplement: Multimedia Appendix 2 [file mhealth_v12i1e54866_app2.pdf]

UNIVERSITÉ DE MONTPELLIER

Direction de la Recherche et des Études Doctorales
Bureau des instances et d'appui réglementaire
Secrétariat du Comité d'Ethique de la Recherche
dred-saisine-cer@umontpellier.fr
04.67.14.30.23

CC 404 - Place Eugène Bataillon
34095 Montpellier Cedex 5

WWW.UMONTPELLIER.FR

Comité d'Éthique de la Recherche de l'Université de Montpellier

Avis consultatif n° UM 2022-006bis

Vu Règlement (UE) 2016/679 du Parlement européen et du Conseil du 27 avril 2016 relatif à la protection des personnes physiques à l'égard du traitement des données à caractère personnel et à la libre circulation de ces données, et abrogeant la directive 95/46/CE,

Vu le Règlement Intérieur du Comité d'Éthique de la Recherche de l'Université de la Recherche,

Les membres du Comité d'Éthique de la Recherche de l'UM entendus en sa séance du 13/07/2022,

Les membres du Comité d'Éthique de la Recherche de l'UM entendus en sa séance du 05/10/2022,

Le Comité d'Éthique de la Recherche de l'Université de Montpellier donne un avis favorable au projet « Évaluation des applications de gestion médicamenteuse sur les plateformes de téléchargements françaises » soumis par MM. Mickaël Toïgo et François Carbonnel.

Fait à Montpellier
Le 7 octobre 2022

Le Président du Comité d'Éthique de la Recherche
de l'Université de Montpellier

Thierry Lavabre-Bertrand
