# Supplementary material for: Quality Assessment of Smartphone Medication Management Apps in France: Systematic Search
Source: JMIR Mhealth Uhealth. 2024 Mar 18;12:e54866. doi: 10.2196/54866 (PMC10985613; doi:10.2196/54866)
Supplement: Multimedia Appendix 3 [file mhealth_v12i1e54866_app3.docx]

**Multimedia Appendix** 3. The full set of scores by application.

| **App** | Platform | Developer | A1 | A2 | A3 | A4 | A5 | **MOY-A** | B6 | B7 | B8 | B9 | **MOY-B** | C10 | C11 | C12 | **MOY-C** | D13 | D14 | D15 | D16 | D17 | D18 | D19 | **MOY-D** | E20 | E21 | E22 | E23 | **MOY-E** | **MARS-F** |
| --- | --- | --- | --- | --- | --- | --- | --- | --- | --- | --- | --- | --- | --- | --- | --- | --- | --- | --- | --- | --- | --- | --- | --- | --- | --- | --- | --- | --- | --- | --- | --- |
| aBox Memo | App Store | ARROW GENERIQUE© | 2 | 2 | 4 | 3,5 | 5 | **3,3** | 3,5 | 5 | 5 | 4,5 | **4,50** | 5 | 3,5 | 3,5 | **4,00** | 5 | 4,5 | 4 | 4 | N/A | 3 | N/A | **4,10** | 4 | 4,5 | 3 | 4 | **3,88** | **3,98** |
| Dosecast: Rappel de ma pilule | App Store | Montuno Software, LLC© | 1,5 | 2 | 3 | 3 | 3 | **2,5** | 5 | 4,5 | 4 | 5 | **4,63** | 4 | 3,5 | 2 | **3,17** | 3,5 | 3 | N/A | N/A | N/A | 3 | 1 | **2,63** | 1 | 1 | 1 | 2 | **1,25** | **3,23** |
| Meddify: Rappel de Pilule | App Store | Oleksandre Bandyliuk© | 2 | 2 | 4 | 4,5 | 5 | **3,5** | 5 | 4 | 5 | 5 | **4,75** | 4,5 | 3,5 | 3 | **3,67** | 3,5 | 3,5 | N/A | N/A | N/A | 1,5 | N/A | **2,83** | 2,5 | 3,5 | 1 | 3 | **2,50** | **3,69** |
| Medical Data Box | App Store | LINKLINKS LTD© | 1,5 | 1,5 | 3 | 4 | 4 | **2,8** | 3 | 3,5 | 3 | 3,5 | **3,25** | 3 | 3 | 2,5 | **2,83** | 5 | 2,5 | N/A | N/A | N/A | 2 | N/A | **3,17** | 2 | 1 | 1 | 2 | **1,50** | **3,01** |
| MedicApp | App Store | AppyCare© | 2 | 2 | 4 | 3,5 | 4,5 | **3,2** | 4,5 | 4 | 4 | 4,5 | **4,25** | 4 | 3 | 2,5 | **3,17** | 5 | 3 | 5 | 4 | N/A | 2 | N/A | **3,80** | 3 | 3 | 1 | 2,5 | **2,38** | **3,60** |
| Medico: Gestion de Médicaments | App Store | Pierre Boudoin © | 2 | 2 | 2 | 2,5 | 4,5 | **2,6** | 4 | 3 | 4 | 4,5 | **3,88** | 4,5 | 3,5 | 3 | **3,67** | 3,5 | 2,5 | 4 | 4,5 | N/A | 2 | N/A | **3,30** | 1,5 | 1 | 1 | 1,5 | **1,25** | **3,36** |
| mediteo rappels de médicaments | App Store | Mediteo GmbH© | 4 | 3,5 | 5 | 5 | 5 | **4,5** | 5 | 5 | 5 | 4,5 | **4,88** | 5 | 3 | 3 | **3,67** | 5 | 5 | 4,5 | 4,5 | N/A | 2,5 | N/A | **4,30** | 5 | 5 | 3 | 5 | **4,50** | **4,34** |
| MedOClock | Apple Store | MedOClock® | 2 | 3 | 4 | 4 | 5 | **3,6** | 2 | 4 | 4,5 | 4,5 | **3,75** | 5 | 3 | 2,5 | **3,50** | 3,5 | 3,5 | N/A | N/A | N/A | 3 | N/A | **3,33** | 2,5 | 3 | 1 | 3 | **2,38** | **3,55** |
| Mon Agenda de Médication | App Store | Baskaran Arunasalam© | 1 | 1,5 | 3,5 | 4 | 4 | **2,8** | 3,5 | 4 | 4 | 4 | **3,88** | 3 | 2 | 1,5 | **2,17** | 4 | 2,5 | N/A | N/A | N/A | 2 | N/A | **2,83** | 1,5 | 1,5 | 1 | 2 | **1,50** | **2,92** |
| PandaLab Ma Santé | App Store | PANDALAB© | 1,5 | 3 | 3,5 | 4,5 | 4,5 | **3,4** | 3,5 | 4,5 | 4,5 | 5 | **4,38** | 4,5 | 4 | 2,5 | **3,67** | 4 | 3 | 4,5 | 4,5 | N/A | 3 | N/A | **3,80** | 2,5 | 3,5 | 1 | 3 | **2,50** | **3,81** |
| Peach : rappel de médicaments | App Store | Happy Peach© | 4 | 3 | 3 | 3,5 | 5 | **3,7** | 3 | 5 | 5 | 4,5 | **4,38** | 4,5 | 4,5 | 4 | **4,33** | 3 | 5 | 4,5 | 4 | N/A | 2 | N/A | **3,70** | 3,5 | 5 | 2 | 4 | **3,63** | **4,03** |
| Pilule: Rappel de médicament | App Store | Wachanga LTD© | 2 | 2 | 3,5 | 4 | 5 | **3,3** | 5 | 4 | 4,5 | 5 | **4,63** | 4,5 | 4 | 3,5 | **4,00** | 4 | 5 | N/A | N/A | N/A | 3 | N/A | **4,00** | 3,5 | 4 | 3 | 4,5 | **3,75** | **3,98** |
| Piule App: Rappel Alarme | App Store | Benjamin Brewis© | 2,5 | 2 | 4,5 | 4 | 5 | **3,6** | 5 | 5 | 5 | 4,5 | **4,88** | 4 | 3,5 | 3 | **3,50** | 4 | 3,5 | N/A | N/A | N/A | 2 | N/A | **3,17** | 4 | 4 | 1,5 | 3,5 | **3,25** | **3,79** |
| Rappel & Réveil de Médicament | App Store | Roman Nikolaev© | 2 | 2 | 4 | 3,5 | 3,5 | **3** | 4 | 4,5 | 4 | 5 | **4,38** | 4 | 3,5 | 3 | **3,50** | 2,5 | 3,5 | N/A | N/A | N/A | 1,5 | N/A | **2,50** | 2,5 | 2,5 | 1 | 2,5 | **2,13** | **3,34** |
| Rappel de la Pilule - Medecine | App Store | Halis Bilal Kara© | 1,5 | 1,5 | 2,5 | 1,5 | 4 | **2,2** | 4 | 4,5 | 4 | 3,5 | **4,00** | 4 | 3 | 2,5 | **3,17** | 2 | 1,5 | N/A | N/A | N/A | 2 | N/A | **1,83** | 1,5 | 1,5 | 1 | 1 | **1,25** | **2,80** |
| Rappel de Médicament | App Store | Aplicativos Legais© | 1 | 1 | 2 | 4 | 4 | **2,4** | 4,5 | 4 | 5 | 5 | **4,63** | 4 | 3,5 | 2,5 | **3,33** | 3 | 3,5 | N/A | N/A | N/A | 1 | N/A | **2,50** | 1 | 1 | 1 | 1,5 | **1,13** | **3,21** |
| Rappel de pilule & médicament | App Store | Whisper Arts© | 2 | 2 | 4 | 3,5 | 4 | **3,1** | 4 | 4 | 4 | 4,5 | **4,13** | 3,5 | 2 | 2 | **2,50** | 3,5 | 3,5 | N/A | N/A | N/A | 2 | N/A | **3,00** | 2,5 | 3 | 1 | 2,5 | **2,25** | **3,18** |
| Rappel de Pilule et Medicament | App Store | MediSafe© | 2 | 2,5 | 5 | 4,5 | 5 | **3,8** | 3,5 | 4 | 4,5 | 5 | **4,25** | 5 | 4 | 3,5 | **4,17** | 4,5 | 5 | N/A | N/A | N/A | 3 | 4 | **4,13** | 4,5 | 5 | 3 | 4,5 | **4,25** | **4,09** |
| Rappel de pilule médicale | App Store | AppAspect Technologies Pvt. LtD. © | 1 | 1,5 | 3 | 2,5 | 2,5 | **2,1** | 2 | 3,5 | 3,5 | 5 | **3,50** | 4 | 2,5 | 2 | **2,83** | 3 | 2 | N/A | N/A | N/A | 1,5 | N/A | **2,17** | 1 | 1 | 1 | 1,5 | **1,13** | **2,65** |
| Rappel de prise de médicaments | App Store | Smiko© | 1,5 | 1,5 | 2,5 | 2 | 4 | **2,3** | 4 | 4 | 4 | 4,5 | **4,13** | 3,5 | 2,5 | 2,5 | **2,83** | 3 | 2,5 | N/A | N/A | N/A | 1,5 | N/A | **2,33** | 1,5 | 1,5 | 1 | 1,5 | **1,38** | **2,90** |
| Rappels de Médicaments | App Store | smartpatient GmbH© | 2,5 | 3 | 4,5 | 5 | 4,5 | **3,9** | 4 | 4,5 | 4,5 | 5 | **4,50** | 5 | 4,5 | 2,5 | **4,00** | 4,5 | 5 | N/A | N/A | N/A | 3 | 4 | **4,13** | 4 | 4,5 | 3 | 4,5 | **4,00** | **4,13** |
| Santé | App Store | Apple© | 2,5 | 2,5 | 3 | 3,5 | 5 | **3,3** | 5 | 4 | 3,5 | 4,5 | **4,25** | 4 | 4,5 | 3 | **3,83** | 4 | 3,5 | 4 | 3,5 | N/A | 2,5 | N/A | **3,50** | 3 | 3,5 | 1 | 3 | **2,63** | **3,72** |
| TOM Rappel medicaments, pilule | App Store | Innovation6 GmbH© | 3 | 3 | 5 | 5 | 4,5 | **4,1** | 5 | 4,5 | 5 | 5 | **4,88** | 4,5 | 4,5 | 3,5 | **4,17** | 5 | 5 | N/A | N/A | N/A | 3 | N/A | **4,33** | 4,5 | 5 | 3 | 4,5 | **4,25** | **4,37** |
| Tracker, Reminder - CareClinic | App Store | CareClinic© | 2,5 | 3 | 3,5 | 4 | 3 | **3,2** | 4 | 2,5 | 3,5 | 4 | **3,50** | 4,5 | 4 | 3,5 | **4,00** | 4 | 4,5 | N/A | N/A | N/A | 3 | 3 | **3,63** | 2 | 1,5 | 1 | 2,5 | **1,75** | **3,58** |
| Yumed | App Store | Yumed© | 2,5 | 2,5 | 4 | 4 | 5 | **3,6** | 3 | 5 | 5 | 5 | **4,50** | 4,5 | 4 | 3 | **3,83** | 3,5 | 3,5 | N/A | N/A | N/A | 2,5 | N/A | **3,17** | 2,5 | 3,5 | 1 | 3 | **2,50** | **3,78** |
| aBox Memo | Google Play | arrow generiques© | 2 | 2 | 4 | 4,5 | 5 | **3,5** | 4 | 5 | 5 | 4,5 | **4,63** | 5 | 3,5 | 3,5 | **4,00** | 4,5 | 4 | 4 | 3,5 | N/A | 3 | N/A | **3,80** | 4 | 5 | 4 | 4,5 | **4,38** | **3,98** |
| Alarme rappel de médicament | Google Play | caiocrol | 1,5 | 1,5 | 3,5 | 3 | 4,5 | **2,8** | 4,5 | 4 | 4 | 4,5 | **4,25** | 4 | 3 | 2,5 | **3,17** | 4,5 | 3 | N/A | N/A | N/A | 2 | N/A | **3,17** | 2 | 2,5 | 1 | 2 | **1,88** | **3,35** |
| ITI Medics | Google Play | ITI Medics © | 2 | 2 | 3,5 | 3 | 5 | **3,1** | 3 | 4 | 4,5 | 4,5 | **4,00** | 4,5 | 4 | 3 | **3,83** | 4 | 3,5 | N/A | N/A | N/A | 1 | N/A | **2,83** | 1 | 2,5 | 1 | 2,5 | **1,75** | **3,44** |
| mediteo rappels de médicaments | Google Play | Mediteo GmbH© | 4 | 4 | 5 | 4,5 | 5 | **4,5** | 5 | 4,5 | 5 | 4,5 | **4,75** | 5 | 3,5 | 3 | **3,83** | 5 | 5 | 4 | 4,5 | N/A | 2,5 | N/A | **4,20** | 5 | 5 | 4 | 5 | **4,75** | **4,32** |
| MedOCLock | Google Play | MedOClock® | 2 | 2 | 3,5 | 4 | 4,5 | **3,2** | 3 | 3 | 4 | 4 | **3,50** | 4,5 | 3 | 3 | **3,50** | 4 | 4 | N/A | N/A | N/A | 3 | N/A | **3,67** | 2,5 | 2,5 | 1 | 3 | **2,25** | **3,47** |
| Mon rappel | Google Play | KMJ Apps | 1 | 1 | 2,5 | 3,5 | 2,5 | **2,1** | 3,5 | 3,5 | 4 | 4,5 | **3,88** | 4 | 2,5 | 2 | **2,83** | 4 | 2,5 | N/A | N/A | N/A | 2 | N/A | **2,83** | 1 | 1,5 | 1 | 2 | **1,38** | **2,91** |
| Peach | Google Play | HappyPeach© | 3,5 | 2,5 | 4 | 4 | 5 | **3,8** | 2 | 4,5 | 3,5 | 4,5 | **3,63** | 4,5 | 4 | 4 | **4,17** | 3 | 5 | 4 | 4 | N/A | 3 | N/A | **3,80** | 1,5 | 1 | 1 | 2 | **1,38** | **3,85** |
| Pill Medicine Reminder | Google Play | Fitness & Entertainment | 1,5 | 1,5 | 3,5 | 3 | 4 | **2,7** | 4,5 | 5 | 4 | 4,5 | **4,50** | 3,5 | 2 | 2 | **2,50** | 3,5 | 3 | N/A | N/A | N/A | 2 | N/A | **2,83** | 2 | 2,5 | 1 | 2 | **1,88** | **3,13** |
| Pills Time Rappel de Pilule | Google Play | Mobile Creatures | 2,5 | 2 | 4 | 3,5 | 4 | **3,2** | 4 | 4,5 | 4,5 | 4 | **4,25** | 4 | 3,5 | 3 | **3,50** | 3 | 4 | N/A | N/A | N/A | 2 | N/A | **3,00** | 2 | 3 | 1 | 3 | **2,25** | **3,49** |
| Pilule: Rappel de médicament | Google Play | Wachanga© | 2 | 2 | 4 | 3,5 | 4,5 | **3,2** | 4,5 | 4 | 5 | 5 | **4,63** | 5 | 4 | 3,5 | **4,17** | 4,5 | 5 | N/A | N/A | N/A | 3 | N/A | **4,17** | 3,5 | 4,5 | 3 | 4,5 | **3,88** | **4,04** |
| Rappel & suivi des médicaments | Google Play | MedicaApp© | 2 | 2,5 | 4 | 4,5 | 5 | **3,6** | 4 | 4 | 4 | 4,5 | **4,13** | 3,5 | 2,5 | 2 | **2,67** | 4,5 | 5 | N/A | N/A | N/A | 2 | N/A | **3,83** | 3 | 3,5 | 2 | 3,5 | **3,00** | **3,56** |
| Rappel de Médicaments | Google Play | Ramtin Software Solutions, LLC | 1,5 | 2 | 4 | 5 | 3 | **3,1** | 4 | 3 | 3,5 | 4,5 | **3,75** | 2 | 2 | 1,5 | **1,83** | 4,5 | 4 | 2 | 2 | N/A | 2 | N/A | **2,90** | 1,5 | 1,5 | 1 | 2,5 | **1,63** | **2,90** |
| Rappel de médicaments | Google Play | Tadpole | 1 | 1 | 2 | 2 | 4 | **2** | 2,5 | 4 | 3,5 | 4 | **3,50** | 4 | 2,5 | 2,5 | **3,00** | 3,5 | 2,5 | N/A | N/A | N/A | 2 | N/A | **2,67** | 1 | 1 | 1 | 1 | **1,00** | **2,79** |
| Rappel de médicaments facile | Google Play | BayRehber / 1KOLAY© | 1 | 1 | 3,5 | 3,5 | 4,5 | **2,7** | 2,5 | 3,5 | 4 | 4,5 | **3,63** | 4 | 3 | 2 | **3,00** | 2,5 | 2,5 | N/A | N/A | N/A | 2 | N/A | **2,33** | 1,5 | 1,5 | 1 | 1,5 | **1,38** | **2,91** |
| Rappel de pilule en français | Google Play | Mikheev Aleksey | 2 | 2 | 3,5 | 3,5 | 4,5 | **3,1** | 4,5 | 4,5 | 5 | 4,5 | **4,63** | 4 | 3,5 | 3 | **3,50** | 4 | 3,5 | N/A | N/A | N/A | 2 | N/A | **3,17** | 2,5 | 2,5 | 1 | 2,5 | **2,13** | **3,60** |
| Rappel de Pilule et Medicament | Google Play | Medisafe© | 2,5 | 3 | 5 | 5 | 4,5 | **4** | 4 | 4 | 4 | 5 | **4,25** | 5 | 4 | 3,5 | **4,17** | 5 | 5 | 3 | 4,5 | 3 | 3 | 4 | **3,93** | 4 | 5 | 4 | 4,5 | **4,38** | **4,09** |
| Rappel de Pilule et Medicament | Google Play | DZMITRY | 2 | 2 | 4 | 2,5 | 4,5 | **3** | 4,5 | 4 | 4 | 4 | **4,13** | 4,5 | 3 | 2,5 | **3,33** | 4,5 | 3,5 | N/A | N/A | N/A | 2 | N/A | **3,33** | 3 | 3,5 | 1 | 3,5 | **2,75** | **3,45** |
| Rappel de pilule et médicament | Google Play | MyTherapy© | 2 | 2,5 | 4,5 | 4,5 | 5 | **3,7** | 5 | 4,5 | 4,5 | 5 | **4,75** | 4,5 | 4 | 2,5 | **3,67** | 4,5 | 4,5 | N/A | N/A | N/A | 3 | 4 | **4,00** | 4 | 5 | 3 | 4,5 | **4,13** | **4,03** |
| Rappel en français | Google Play | F. Zander | 1 | 1 | 2 | 3 | 2,5 | **1,9** | 3 | 2,5 | 4 | 4 | **3,38** | 4 | 3 | 2,5 | **3,17** | 4 | 2,5 | N/A | N/A | N/A | 2 | N/A | **2,83** | 1 | 1 | 1 | 1,5 | **1,13** | **2,82** |
| Rappel pillule et médicament | Google Play | Whisper Arts© | 2 | 2 | 4,5 | 4 | 4 | **3,3** | 4,5 | 3 | 4 | 5 | **4,13** | 3 | 3 | 2 | **2,67** | 3,5 | 4,5 | N/A | N/A | N/A | 2 | N/A | **3,33** | 2 | 3 | 1 | 3 | **2,25** | **3,36** |
| Remède Temps! | Google Play | JMSoft Applications© | 2 | 2 | 3,5 | 3 | 3,5 | **2,8** | 4 | 3,5 | 4,5 | 5 | **4,25** | 4,5 | 3 | 3 | **3,50** | 3,5 | 3,5 | N/A | N/A | N/A | 1,5 | N/A | **2,83** | 2 | 2,5 | 1 | 2 | **1,88** | **3,35** |
| RxDroid | Google Play | Joseph C. Lehner | 2 | 1,5 | 4 | 3 | 4,5 | **3** | 4,5 | 4 | 4,5 | 5 | **4,50** | 4,5 | 3 | 2,5 | **3,33** | 4 | 3,5 | N/A | N/A | N/A | 2 | N/A | **3,17** | 2,5 | 3 | 1 | 3 | **2,38** | **3,50** |
| TOM | Google Play | Innovation 6© | 3 | 3 | 4,5 | 4,5 | 5 | **4** | 5 | 4 | 5 | 4,5 | **4,63** | 5 | 3,5 | 3,5 | **4,00** | 5 | 5 | N/A | N/A | N/A | 3 | N/A | **4,33** | 5 | 5 | 4 | 5 | **4,75** | **4,24** |
| Yumed - rappels de médicaments | Google Play | Yumed© | 2 | 2 | 4 | 3,5 | 5 | **3,3** | 3 | 5 | 5 | 5 | **4,50** | 4,5 | 4 | 3 | **3,83** | 3,5 | 3 | N/A | N/A | N/A | 2,5 | N/A | **3,00** | 3 | 3,5 | 1 | 3 | **2,63** | **3,66** |
