# Supplementary material for: Quality Assessment of Smartphone Medication Management Apps in France: Systematic Search
Source: JMIR Mhealth Uhealth. 2024 Mar 18;12:e54866. doi: 10.2196/54866 (PMC10985613; doi:10.2196/54866)
Supplement: Multimedia Appendix 4 [file mhealth_v12i1e54866_app4.docx]

**Multimedia Appendix 4.** The MARS-F ranking of apps by platform.

| **Application** | **Plateforme** | **Développeur** | **MARS-F** |
| --- | --- | --- | --- |
| TOM Rappel medicaments. pilule | App Store | Innovation6 GmbH© | **4.37** |
| mediteo rappels de médicaments | App Store | Mediteo GmbH© | **4.34** |
| Rappels de Médicaments | App Store | smartpatient GmbH© | **4.13** |
| Rappel de Pilule et Medicament | App Store | MediSafe© | **4.09** |
| Peach : rappel de médicaments | App Store | Happy Peach© | **4.03** |
| Pilule: Rappel de médicament | App Store | Wachanga LTD© | **3.98** |
| aBox Memo | App Store | ARROW GENERIQUE© | **3.98** |
| PandaLab Ma Santé | App Store | PANDALAB© | **3.81** |
| Piule App: Rappel Alarme | App Store | Benjamin Brewis© | **3.79** |
| Yumed | App Store | Yumed© | **3.78** |
| Santé | App Store | Apple© | **3.72** |
| Meddify: Rappel de Pilule | App Store | Oleksandre Bandyliuk© | **3.69** |
| MedicApp | App Store | AppyCare© | **3.60** |
| Tracker. Reminder - CareClinic | App Store | CareClinic© | **3.58** |
| MedOClock | App Store | MedOClock® | **3.55** |
| Medico: Gestion de Médicaments | App Store | Pierre Boudoin © | **3.36** |
| Rappel & Réveil de Médicament | App Store | Roman Nikolaev© | **3.34** |
| Dosecast: Rappel de ma pilule | App Store | Montuno Software. LLC© | **3.23** |
| Rappel de Médicament | App Store | Aplicativos Legais© | **3.21** |
| Rappel de pilule & médicament | App Store | Whisper Arts© | **3.18** |
| Medical Data Box | App Store | LINKLINKS LTD© | **3.01** |
| Mon Agenda de Médication | App Store | Baskaran Arunasalam© | **2.92** |
| Rappel de prise de médicaments | App Store | Smiko© | **2.90** |
| Rappel de la Pilule - Medecine | App Store | Halis Bilal Kara© | **2.80** |
| Rappel de pilule médicale | App Store | AppAspect Technologies Pvt. LtD. © | **2.65** |
|  |  |  |  |
| mediteo rappels de médicaments | Google Play Store | Mediteo GmbH © | **4.32** |
| TOM | Google Play Store | Innovation 6 (AG) © | **4.24** |
| Rappel de Pilule et Medicament | Google Play Store | Medisafe © | **4.09** |
| Pilule: Rappel de médicament | Google Play Store | Wachanga © | **4.04** |
| Rappel de pilule et médicament | Google Play Store | MyTherapy © | **4.03** |
| aBox Memo | Google Play Store | arrow generiques © | **3.98** |
| Peach | Google Play Store | HappyPeach © | **3.85** |
| Yumed - rappels de médicaments | Google Play Store | Yumed © | **3.66** |
| Rappel de pilule en français | Google Play Store | Mikheev Aleksey | **3.60** |
| Rappel & suivi des médicaments | Google Play Store | MedicaApp © | **3.56** |
| RxDroid | Google Play Store | Joseph C. Lehner | **3.50** |
| Pills Time Rappel de Pilule | Google Play Store | Mobile Creatures | **3.49** |
| MedOCLock | Google Play Store | MedOClock® | **3.47** |
| Rappel de Pilule et Medicament | Google Play Store | DZMITRY | **3.45** |
| ITI Medics | Google Play Store | ITI Medics © | **3.44** |
| Rappel pillule et médicament | Google Play Store | Whisper Arts © | **3.36** |
| Alarme rappel de médicament | Google Play Store | caiocrol | **3.35** |
| Remède Temps! | Google Play Store | JMSoft Applications © | **3.35** |
| Pill Medicine Reminder | Google Play Store | Fitness & Entertainment | **3.13** |
| Rappel de médicaments facile | Google Play Store | BayRehber / 1KOLAY | **2.91** |
| Mon rappel | Google Play Store | KMJ Apps | **2.91** |
| Rappel de Médicaments | Google Play Store | Ramtin Software Solutions. LLC | **2.90** |
| Rappel en français | Google Play Store | F. Zander | **2.82** |
| Rappel de médicaments | Google Play Store | Tadpole | **2.79** |
