# Supplementary material for: Quality Assessment of Smartphone Medication Management Apps in France: Systematic Search
Source: JMIR Mhealth Uhealth. 2024 Mar 18;12:e54866. doi: 10.2196/54866 (PMC10985613; doi:10.2196/54866)
Supplement: Multimedia Appendix 5 [file mhealth_v12i1e54866_app5.docx]

**Multimedia Appendix 5.** User ratings and number of evaluation of apps by platform.
